# Nutrition Promotion to Prevent Obesity in Young Adults

**DOI:** 10.3390/healthcare3030809

**Published:** 2015-09-07

**Authors:** Margaret A. Allman-Farinelli

**Affiliations:** School of Molecular Bioscience, Charles Perkins Centre, D17, University of Sydney, Sydney 2006, Australia; E-Mail: margaret.allmanfarinelli@sydney.edu.au; Tel.: +61-2-90367045

**Keywords:** nutrition, preventive healthcare, obesity, young adults, behavior change, food environments

## Abstract

Young adulthood is a vulnerable period for weight gain and the health consequences of becoming obese during this life-stage of serious concern. Some unhealthy dietary habits are typical of young adults in many developed nations encountering the obesity epidemic. These include high sugar-sweetened beverage consumption, lower vegetable intake and greater consumption of foods prepared outside the home including fast foods. Each of these dietary behaviours may place young adults at increased risk for overweight and obesity. Evidence suggests many young adults with unhealthy nutrition behaviours are not considering nor preparing to make changes. To improve their nutrition and health as they progress through the lifecycle requires approaches specifically targeted to this age group. Strategies and programs should include both individual level and population approaches. The evidence base for prevention of weight gain and halting overweight and obesity in young adulthood is currently small with few studies of high quality. Studies modifying food environments in colleges and universities are also of limited quality, but sufficiently promising to conduct further research employing better, more sophisticated, study designs and additionally to include health outcome measures. More research into programs tailored to the needs of young adults is warranted with several studies already underway.

## 1. Introduction

For more than three decades, the developed world has experienced an obesity epidemic with little evidence to indicate prevalence is declining [1]. Concerted efforts have had a strong focus on childhood prevention programs with the realization that in a country such as the US, one in three children was overweight or obese [2]. Programs for the management of obesity have encompassed all age groups. However, they have usually had a stronger emphasis on the middle-aged given that prevalence is highest for this demographic e.g., almost 40% of 40 to 59 year-olds in the US are obese [2]. Some OECD countries such as the UK and USA are beginning to show a stabilization of rates but in countries like Australia and Mexico the prevalence continues to increase [1].

There is now awareness that young adulthood is a period of rapid weight gain, and that the mean body mass index of the youngest generation as they enter adulthood becomes higher than the preceding generation [3,4], with some variation between countries. For example, in Ireland it has been reported that while age and period give rise to increases in obesity, recency of birth gives a slightly protective advantage [5]. In Australia, the young adults are becoming fatter sooner [6]. The phenomenon of the “Freshman Fifteen”, referring to the average weight gain when a young adult starts college in the US, is well documented [7]. There is a growing awareness that much is to be gained by preventing weight increase in particular, deposition of visceral fat that leads to metabolic distress and chronic disease [8]. Preventing overweight young adults from becoming obese should be a priority in the management of the obesity epidemic.

The genesis of the obesity epidemic in the US and much of the Western world began more than 30 years ago. At this time the food supply began to change with a greater availability of relatively inexpensive highly processed foods that could be high in saturated fat, sugars and starches with high energy density [9]. In addition, the consumption of fast food and other meals prepared outside the home has risen. The current generation of young adults, aged 18 to 35 years, were born into this “obesogenic food environment” and therefore have only ever experienced this food culture.

This review will discuss the health consequences of becoming overweight and obese at younger ages defined as 18 to 35 years; the individual dietary behaviours that define younger adults; their readiness to change behaviours and strategies that might be employed to enable better quality diets and prevention of weight gain. The appropriate media to disseminate nutrition promotion at the individual level of behavior change and at the public health and food environment policy level will be discussed.

## 2. Background

Early onset of obesity has been demonstrated to lead to chronic illness development at a younger age and increase all-cause mortality. Cohort studies from the US, UK and Japan indicate that those who have BMI of 25 kg/M^2^ or greater during young adulthood are more likely to have a premature death [10,11,12]. Weight gain between 20 and 35 years was demonstrated to predict all-cause mortality between 50 and 69 years of age in the US cohort [10]. Causes of death include cardiovascular disease but also stroke and cancers. Taing *et al.*, using data from the US Women’s Health Initiative, reported mortality risk increased with every 5 kg of weight gained during early (hazard ratio (HR) = 1.04 (1.00–1.07)) or mid-adulthood (HR = 1.05 (1.02–1.08)) [13]. It is suggested that individuals who gain weight and become overweight and obese earlier have a longer exposure to experience the adverse metabolic effects and consequent disease. While treatments for cardiovascular disease continue to improve the human and economic costs for other diseases resulting from obesity, such as diabetes and its complications, and cancers might likely rise. For example, in 2009 Aitken *et al*. reported the projected costs of obesity-related hospitalisations in those born 1970 to 1980 would be more than double those for a cohort born 20 years earlier. This was because they would experience a higher incidence of obesity across the life cycle [14].

## 3. Individual Food Behaviours Distinctive of Young Adults

Most western nations have shown changes in the nutritional composition of the diet over the past three to four decades. The most obvious change has been a decline in the percentage energy from fat and an increase in percentage energy from carbohydrate. As more was learnt about the role of saturated fat and serum cholesterol, a switch away from fat occurred, with replacement by carbohydrates. The change in nutrient composition is reflected in the diets of all age groups including the young adult. For example, in Australia 19 to 30 year olds and 31 to 50 year olds both consume 31% energy from fat and 18% from protein [15]. While it appears populations may have achieved the original goal set for total fat intakes to decrease cardiovascular disease risk, the intake of saturated fat remains above that recommended and unsaturated fat lower than suggested. Replacement of unsaturated fat with carbohydrate is undesirable.

However, young adults demonstrate some distinctive food and beverage behaviours in most developed nations grappling with obesity. Among adults, it is the youngest that have highest consumption of sugar-sweetened beverages in the US, UK, New Zealand and Australia [15,16,17,18]. With a growing awareness that the energy derived from liquids, such as soft drinks, may not be compensated by a reduction in calories from food intake and their association with other poor health outcomes, such as dental caries, cardiovascular disease and diabetes, the findings for sugary drink intake are of concern [19,20,21]. Trends in vegetable consumption show younger adults to be the poorest consumers, for example, in France [22] and Australia [15]. While the evidence that vegetable consumption prevents weight gain is somewhat equivocal it is well recognized that vegetables are a major dietary source of beta-carotene, potassium, folate, soluble fibre and anti-oxidants and constitute part of a healthy diet [23]. Young adults also have the greatest intakes of food prepared outside the home [15,24,25]. Studies have indicated that consumption of fast food two or more times weekly is associated with increases in waist circumference (*i.e*., abdominal obesity) in young adults in Australia [26]. The CARDIA study in the US reported that the average 15 year weight gain in those who ate at a fast food outlet less than once per week was 4.5 kg less than for those who ate there more than twice per week (*p* = 0.005) and their insulin resistance was halved (*p* = 0.008) [27]. Findings from a review of prospective studies in all age groups for an association between changes in BMI and eating food prepared away from home show inconsistencies, but warrant continued investigation [28].

Some differences are observed by gender and socioeconomic status with respect to these nutrition behaviours. In the US those young adults with lower education drink more sugar-sweetened beverages but global statistics show young adults in countries with upper middle-incomes drink more than low income countries and males drink more than females [29,30]. Vegetable intakes were similar by gender in Australia and France but have previously been shown to demonstrate a difference by socioeconomic status [31]. Consumption of take-away foods may be greater among those of lower socioeconomic status [31] but this is not a universal finding [25,32].

Evidence of a causal relationship between these three diet-related behaviours and weight gain requires further confirmation. However, it remains of interest that sugary drinks, fast food and poor vegetable intake consistently feature in young adults dietary habits. Vegetables are one food that lowers the overall energy-density of a diet, while sugary drinks and fast food substantially raise it. In a 20 year follow-up of the CARDIA cohort, recruited at ages 18 to 30 years in 1985–1986, it was demonstrated that weight gain and lack of cardiorespiratory fitness, but not macronutrient composition of the diet distinguished those who became the metabolically healthy overweight/obese [33]. Furthermore, the risk of metabolic syndrome in CARDIA participants was lowered with adherence to a diet rich in fruit, vegetables, whole grains, nuts and fish [34].

## 4. Readiness of Young Adults to Change Behaviours and Enablers and Barriers

The question now arises as to how ready young adults are to change the afore-mentioned nutrition behaviours. The Transtheoretical Model of behavior change reasons that adults demonstrate their readiness for change via five stages; precontemplation which means the subjects are not yet considering change; contemplation which means participants are considering change; in the preparation stage subjects are getting ready to change their behaviours and then move to the next stage of action. Those who have practiced the behavior for an extended period such as six months may be considered in maintenance [35]. In a study of 2024 low-income adults aged ages 18–24, Nitzke *et al*., reported 62.4% were in pre-action stages for vegetable intake [36]. Kattlemann *et al*., found that only 12% of a college sample of 1639 young adults consumed five cups or more of fruit and vegetables per day, but 43.8% were in a pre-action stage-of-readiness-to-change [37]. A small sample of predominantly younger Australians (mean age 33 years) were asked about which of three nutrition behaviours, fruit and vegetable, fat or sugar-sweetened beverages intake they would consider changing. Two-thirds of those with inadequate fruit and vegetables intended to improve intakes, three out of five to change fat and two-fifths to change their beverage habit [38]. It was found that for fruit and vegetables consumption 30% of participants were still in precontemplation. However, for those 57% of participants in contemplation or preparation, they demonstrated they were more conscious of their behavior, were somewhat concerned by it (dramatic relief), believed they had the ability to improve intake (self-liberation) and that being healthy was important (self-reevaluation) to who they were [38]. Wyker *et al*., reported on a sample of 204 college students finding that 92% were still in the precontemplation stage for eating five serves of fruit and vegetables per day [39].

Hattersley *et al*., reported that even when young adults were aware of the link between sugar-sweetened beverages and poor health outcomes they did not see it as personally relevant because the time course of disease development was very much in the future [40]. Consumption of sugar-sweetened beverages was seen as a social norm and important when socializing with friends [41]. Females were more concerned than males with many switching to fruit juices and unaware of the energy and sugars they provided [40].

The Theory of Planned Behaviour suggests that the individual’s attitude, social norms and intentions together with perceived behavioural control or “self-efficacy” to perform the behavior will predict how one changes a behavior [42]. A study in the UK of 18 to 25-year-olds found that 40% believed they were eating sufficient fruit and vegetables and 59% regular meals but the main problem with their diet was unhealthy snacking and while 89% had a high intention for having a healthy diet it did not translate into action [43]. A study in Norway of 519 young adults assessed the constructs of the Theory of Planned Behavior at age 25 years and subsequent intake 8 years later for four nutrition-related behaviours (intake of fruits and vegetables, whole grains, total fat and added sugar). It showed that all constructs intention, attitudes, subjective norms, perceived behavioural control, and perceived social norms were all predictive of one or more behaviours with differences between genders and nutrition behaviours [44].

Thus while readiness to change is variable, and attitudes predict subsequent intakes, it remains important to raise awareness about the risks of these deleterious nutrition behaviours and plan appropriate programs for change.

## 5. Programs for Individual Behavior Change

The evidence base for interventions targeting prevention of obesity in young adults is small and the studies usually of a poor to moderate quality with small sample sizes. However, it seems that interventions involving change in nutrition and/or physical activity behaviours are producing modest weight loss in the short term. [45,46,47]. Program elements that appear to support change include dietary guidance, advice on physical activity, ongoing support be it face-to-face, phone or online with tailoring of advice and self-monitoring of weight and/or lifestyle behaviours. Among the limitations is that most studies fail to assess or report factors that would permit them to be translated and scaled up and implemented in the community-at-large [47]. Few researchers detail how to engage the population and recruit mostly females, and almost none of them assess cost-effectiveness nor evaluate the entire process of development and implementation of the trials [47].

There are a number of well-designed randomised controlled trials nearing completion in the US and others in progress in countries such as Australia. Seven institutions in the US have been funded by the *Eunice Kennedy Shriver* National Institute of Child Health and Human Development (NICHD) together with the National Heart Lung Blood Institute to conduct the Early Adult Reduction of weight through LifestYle intervention (EARLY) Trials (see Table 1). All participants must be aged between 18 and 35 years with a BMI between 20 and 40 kg/M2 (although ≥18.5 is the lower limit in the pregnancy study). Each of these focuses on either weight loss or prevention of weight gain and one addresses prevention of excessive weight gain in pregnancy. Change in weight or BMI is the primary outcome in all studies. These interventions are 24 months in duration and incorporate media and technologies frequently used by the age group [48]. The interventions use different delivery modes including web-based courses, text messaging, Smartphone apps and social media and self-monitoring devices.

There are a number of other registered trials underway in other countries, in particular, Australia. These include a mobile lifestyle program for 18 to 35 year olds [49] and a program with mobile devices and text messaging to improve nutrition [50]. In addition, a multidisciplinary lifestyle intervention on cardiovascular risk factors for 18 to 30 year olds [51], and a program for weight loss of 5% to 10% in young women is registered [52] (see Table 2). Preliminary findings for the “TXT2BFiT” study indicated that the 12-week program resulted in a 2.2 kg (95% CI 0.8–3.6) difference in weight between the intervention and control group and improved intakes of vegetables with decreased intake of sugar-sweetened beverages and take-away foods [53]. Share *et al*., reported that there were no differences between the intervention group and the wait-list control group other than physical activity but this was due to decreases in waist circumference on both groups [54]. The intervention group maintained the benefit at 24 weeks [54].

**Table 1:** Outline summary of the Early Adult Reduction of weight through LifestYle intervention (EARLY) trials being conducted in the US [48].

Principal Investigator	Study Name	Population	Sample Size	Intervention	Comparison	Outcome
Leslie Lytle University of Minnesota	CHOICES	Community College students	441	One credit college course on behaviours for weight control. Web-based social network site with goal setting and tracking of weight and behaviours	Public Health information only and usual care	Change in BMI
Laura Svetkey Duke University	CITY	Overweight/obese young adults	365	Two intervention arms (1) Cell phone self-monitoring (2) Cell phone plus individual and group coaching	Usual Care	Change in weight
Christine Olsen Cornell University & Isabel Fernandez University of Rochester	e-MomsRoc	Pregnant women	1691	(1) Intervention during pregnancy (2) Intervention during pregnancy and post-partum Intervention via cell phone and web site; goal setting, self-monitoring & incentives; action-oriented behavioural messages	Non-weight related information on web site	Difference in proportion unhealthy gestational weight gain and weight retention post-partum
John Jakicic University of Pittsburgh	IDEA	Overweight/obese young adults	471	Standard plus Enhanced weight loss intervention. Additional treatment are text messages, self- monitoring via web site plus wearable monitor to track activity/energy expenditure	Control Standard weight loss intervention; face-to-face plus phone calls	Change in weight
Kevin Patrick University of California, San Diego	SMART	Overweight/obese 4 year college students	404	Intervention theory based content on physical activity diet and weight management via text messages, emails Facebook and Apps	Control web site with standard health information	Change in weight
Rena Wing Brown University Deborah Tate University of North Carolina	SNAP	Young adults	600	(1) Large changes intervention in diet and physical activity to lose 5 to 10l b (2) Small changes intervention for diet and physical activity to avoid weight gain	Usual care	Change in weight
Karen Johnson University of Tennessee	TARGIT	Young adult smokers	330	Tobacco quite line plus Behavioural weight gain prevention program with smoking cessation apps, self-monitoring, webinars and web site	Tobacco quit line	Change in weight

**Table 2:** Registered clinical trials in Australia in young adults.

Principal Investigator	Study Name	Population	Sample Size	Intervention	Comparison	Outcome
Margaret Allman-Farinelli University of Sydney [49]	TXT2BFiT	Overweight young adults 18 to 35 years	250	Lifestyle behavioural intervention with text messages, 5 coaching calls, email, apps and web site for self-monitoring and diet booklet.	4 text messages 1 phone call Public health nutrition and physical activity guidelines	Change in weight
Deborah Kerr Curtin University [50]	CHAT	18 to 30 year olds	300	Two intervention arms. Mobile dietary food record (1) Text messages plus tailored feedback (2) Tailored feedback only	Control arm Mobile dietary food record only	Change in fruit and vegetables intake
Bianca Share Australian Catholic University [51]	12 week multidisciplinary lifestyle intervention	18 to 30 year old women with abdominal obesity	68	Physical activity sessions, nutrition education and cognitive behavioral therapy	Wait-list control	Waist circumference
Melinda Hutchesson University of Newcastle [52]	Be Positive Be Healthe	18 to 35 year old women Overweight/obese	114	Individual advice and goal setting for energy intake and expenditure e-tools web site, apps, text messages, newsletters	Wait list control	Weight change

## 6. Medium for Program Delivery

Kelly *et al*., conducted a systematic literature review identifying 14 dietary interventions for college students [55]. Among the delivery style were face-to-face in classrooms, and online web-based interventions and three were food environment interventions. Only six of the 14 studies selected were randomized controlled trials with five reporting positive changes, but the durations of both intervention and follow-up were mostly very short. The review authors conclude that the web might be an effective intervention medium and recommend that text messaging, and Smartphone applications (apps) be explored [55]. Many of the trials cited above are researching the use of technology. It has already been demonstrated that text messaging is effective in weight management in general adult populations, and this may include changes in nutrition [56,57]. Efficacy testing of Smartphone apps in nutrition promotion is still in its early phase, but research in this area will likely experience exponential growth [58]. Young adults have the largest ownership of Smartphones [59].

## 7. Environmental Level Changes

Nutrition promotion should not be limited to the individual if we are to prevent obesity in young adults but also needs to include changes in the food environments with which they interact. Settings to change the food environment include colleges, universities and workplaces and retail sectors for change include supermarkets, cafes and food malls. Changes in the latter will benefit not only young adults but the entire community. Vending machines have also been singled out as a vehicle for change because they mostly sell sugar-sweetened beverages and energy-dense foods rich in saturated fat, sugar and sodium [60]. Among the most researched strategies for influencing food choices is the provision of nutrition information at point-of-purchase [61]. These include but are not restricted to, energy/calorie labelling, nutrient profiling, and interpretive nutrition labelling, e.g., traffic lights for red foods to avoid, green to encourage consumption. The findings for labelling of food packaging or on menu boards are equivocal but with some showing increased sales of healthy foods and selections lower in energy, fat or sugar. Selected studies have specifically targeted young adults as the primary population of interest, e.g., in tertiary education facilities, but other interventions have been conducted in workplaces and fast food restaurants aimed at all consumers. Some studies show that social marketing and educational programs are important adjuncts if the labeling program is to be successful [61,62].

Other types of intervention in the food environment are to change the availability of unhealthy and healthy food items or to change the pricing structures. If only healthy choices are available, e.g., in vending machines on a college campus, then young adults will select these foods and beverages. Some studies show that making healthy options less expensive while placing taxes on unhealthy foods can bring about positive changes in consumption [63]. Intervention that dispels the belief that eating healthily leads to higher food costs might also prove successful [64].

When considered in its entirety the evidence for food environmental changes is problematic because of poor study design and validity of measurements [65,66]. The complexities of engaging all stakeholders to co-operate must be considered. Fears about falling profits for food supplier, retail outlet or a student body that might benefit financially from sales prevail. Cluster and randomized clinical trials are rare and often the pre and post-intervention design is all that can be accomplished. For this reason, it becomes difficult for governments to implement policy when results remain questionable. Most studies do not include end-points of health such as changes in body weight or even positive changes in the total diet when such interventions are conducted. Policies have been implemented in cities like New York in the US and in New South Wales, Australia that energy labels must be displayed in fast food outlets, cafes and restaurants [67]. However, reports of the prevalence of overweight and obesity in young (or any other) adults declining are not apparent. Sceptics will say restricting sales in one venue, e.g., a college, may only result in the young adult students accessing the food elsewhere. Without evidence, little can be recommended to change the food environment. Study designs that include mapping and auditing food environments must also be mindful that young adults, in particular, are highly mobile and encounter many food environments outside their immediate community [68].

## 8. Other Considerations

While this review has focused on food and nutrition, physical activity and sedentary behavior must always be considered in the context of prevention of weight gain. Life transitions during young adulthood have been demonstrated to contribute to females becoming inactive as they start work, marry and have children, e.g., in Australia [69]. Adolescence to young adulthood declines in moderate to vigorous activity in males are frequent e.g., in Norway [70]. Despite this young adults participate in more moderate to vigorous activity than older adults as demonstrated by the NHANES data in the US [71].

## 9. Conclusions

Younger adults are gaining excessive amounts of weight in many countries with obesity epidemics. It is important that this is recognized, and prevention strategies mobilized, or they face premature chronic disease, disability and death. The evidence base for effective health promotion to cater to this demographic is limited but growing, and should become the next focal point after childhood interventions.
